# Reconsidering *Mycobacterium bovis* as a proxy for zoonotic tuberculosis: a molecular epidemiological surveillance study

**DOI:** 10.1016/S2666-5247(20)30038-0

**Published:** 2020-06

**Authors:** Shannon C Duffy, Sreenidhi Srinivasan, Megan A Schilling, Tod Stuber, Sarah N Danchuk, Joy S Michael, Manigandan Venkatesan, Nitish Bansal, Sushila Maan, Naresh Jindal, Deepika Chaudhary, Premanshu Dandapat, Robab Katani, Shubhada Chothe, Maroudam Veerasami, Suelee Robbe-Austerman, Nicholas Juleff, Vivek Kapur, Marcel A Behr

**Affiliations:** a Department of Microbiology and Immunology, McGill University, Montreal, QC, Canada; b McGill International Tuberculosis Centre, McGill University, Montreal, QC, Canada; c Department of Medicine, McGill University, Montreal, QC, Canada; d Infectious Diseases and Immunity in Global Health Program, Research Institute of the McGill University Health Centre, Montreal, QC, Canada; e Department of Animal Science and the Huck Institutes of the Life Sciences, The Pennsylvania State University, University Park, PA, USA; f National Veterinary Services Laboratories, Animal and Plant Health Inspection Service, US Department of Agriculture, Ames, IA, USA; g Department of Clinical Microbiology, Christian Medical College Vellore, Vellore, India; h Department of Veterinary Public Health and Epidemiology, College of Veterinary Sciences, Lala Lajpat Rai University of Veterinary and Animal Sciences, Hisar, India; i Department of Animal Biotechnology, College of Veterinary Sciences, Lala Lajpat Rai University of Veterinary and Animal Sciences, Hisar, India; j Eastern Regional Station, Indian Veterinary Research Institute, Indian Council of Agricultural Research, Kolkata, India; k Cisgen Biotech Discoveries, Chennai, India; l Bill & Melinda Gates Foundation, Seattle, WA, USA

## Abstract

**Background:**

Zoonotic tuberculosis is defined as human infection with *Mycobacterium bovis*. Although globally, India has the largest number of human tuberculosis cases and the largest cattle population, in which bovine tuberculosis is endemic, the burden of zoonotic tuberculosis is unknown. The aim of this study was to obtain estimates of the human prevalence of animal-associated members of the *Mycobacterium tuberculosis* complex (MTBC) at a large referral hospital in India.

**Methods:**

We did a molecular epidemiological surveillance study of 940 positive mycobacteria growth indicator tube (MGIT) cultures, collected from patients visiting the outpatient department at Christian Medical College (Vellore, India) with suspected tuberculosis between Oct 1, 2018, and March 31, 2019. A PCR-based approach was applied to subspeciate cultures. Isolates identified as MTBC other than *M tuberculosis* or as inconclusive on PCR were subject to whole-genome sequencing (WGS), and phylogenetically compared with publicly available MTBC sequences from south Asia. Sequences from WGS were deposited in the National Center for Biotechnology Information Sequence Read Archive, accession number SRP226525 (BioProject database number PRJNA575883).

**Findings:**

The 940 MGIT cultures were from 548 pulmonary and 392 extrapulmonary samples. A conclusive identification was obtained for all 940 isolates; wild-type *M bovis* was not identified. The isolates consisted of *M tuberculosis* (913 [97·1%] isolates), *Mycobacterium orygis* (seven [0·7%]), *M bovis* BCG (five [0·5%]), and non-tuberculous mycobacteria (15 [1·6%]). Subspecies were assigned for 25 isolates by WGS, which were analysed against 715 MTBC sequences from south Asia. Among the 715 genomes, no *M bovis* was identified. Four isolates of cattle origin were dispersed among human sequences within *M tuberculosis* lineage 1, and the seven *M orygis* isolates from human MGIT cultures were dispersed among sequences from cattle.

**Interpretation:**

*M bovis* prevalence in humans is an inadequate proxy of zoonotic tuberculosis. The recovery of *M orygis* from humans highlights the need to use a broadened definition, including MTBC subspecies such as *M orygis*, to investigate zoonotic tuberculosis. The identification of *M tuberculosis* in cattle also reinforces the need for One Health investigations in countries with endemic bovine tuberculosis.

**Funding:**

Bill & Melinda Gates Foundation, Canadian Institutes for Health Research.

## Introduction

Globally, tuberculosis is the most deadly disease arising from a single infectious agent, leading to 1·4 million deaths each year, primarily in low-income and middle-income countries (LMICs).^1^ Tuberculosis in cattle (bovine tuberculosis) is also a considerable animal health problem and endemic in most LMICs, costing an estimated US$3 billion worldwide each year.^2^ WHO, the World Organisation for Animal Health, and the Food and Agriculture Organization of the United Nations define zoonotic tuberculosis as human infection with *Mycobacterium bovis,* a member of the *Mycobacterium tuberculosis* complex (MTBC).^1^, ^3^, ^4^ As such, detection of *M bovis* is often used as a proxy for measuring the prevalence of zoonotic tuberculosis. On the basis of this definition, WHO estimated that the annual number of human tuberculosis cases due to zoonotic tuberculosis was 143 000 in 2018.^1^

By 2035, WHO is aiming to reduce the incidence of tuberculosis by 90% as a part of its End TB Strategy.^5^ India has the largest burden of human tuberculosis globally, with more than 2·6 million cases and 400 000 deaths reported in 2019.^1^ The cattle population in India exceeds 300 million, a population larger than any other country.^6^ Yet in India, bovine tuberculosis is both uncontrolled and endemic, with an estimated 21·8 million infected cows as of 2017.^7^ Some previous studies have estimated the prevalence of zoonotic tuberculosis in India to be around 10%; however, these studies were limited in their ability to differentiate between MTBC members.^8^, ^9^, ^10^ Other evidence in the past decade has suggested that *Mycobacterium orygis,* an MTBC member described in 2012, might be endemic to south Asia;^11^, ^12^, ^13^, ^14^ however, robust estimates of *M orygis* prevalence in humans and cattle are lacking.

In the present study, we sought to evaluate the definition of zoonotic tuberculosis, and investigate the prevalence of MTBC members in a large referral hospital in southern India, via molecular analyses of 940 positive broth cultures. Our findings are subsequently evaluated within the framework of other sequences collected in south Asia. We sequence 25 isolates by whole-genome sequencing (WGS) and compare these sequences with representative genomes of the MTBC lineages circulating worldwide, and with 715 publicly available genomes collected from cattle and humans in south Asia.

**Research in context**

**Evidence before this study**

WHO, the World Organisation for Animal Health, and the Food and Agriculture Organization of the United Nations currently define zoonotic tuberculosis as human infection with *Mycobacterium bovis* of animal origin. However, other members of the *Mycobacterium tuberculosis* complex (MTBC), such as *Mycobacterium orygis*, have been reported as the cause of tuberculosis in humans, especially in patients from India. Consequently, the burden of zoonotic tuberculosis might be underestimated by surveillance studies restricted to *M bovis*. In India, which has the largest number of human tuberculosis cases and the largest cattle population worldwide, and where bovine tuberculosis is endemic, the burden of zoonotic tuberculosis is unknown. On July 17, 2019, we searched PubMed using the terms “zoonosis” AND “tuberculosis” OR “zoonotic tuberculosis” AND “India” without date or language restrictions, which returned 45 results. Only three studies had screened for zoonotic tuberculosis in humans, none of which used methods capable of reliably differentiating between MTBC subspecies. These three studies were also small in scale (maximum of 331 samples), and none subspeciated positive cultures or attempted to identify zoonotic subspecies other than *M bovis.*

**Added value of this study**

This study is the first to screen pulmonary and extrapulmonary culture isolates in India with tests to detect different MTBC subspecies capable of causing zoonotic tuberculosis. This study represents an advance from previous surveillance reports in that screening was done directly on broth cultures with a two-step protocol, involving identification by PCR, and confirmatory testing by whole-genome sequencing. Our findings were further validated by comparisons in the context of other publicly available sequences collected from humans and cattle from south Asia. Our research showed that in a large referral hospital in southern India, *M bovis* infection is uncommon, and that zoonotic tuberculosis might instead be caused by *M orygis* and potentially *M tuberculosis*.

**Implications of all the available evidence**

The findings indicate that *M bovis* is an inadequate proxy for the detection of zoonotic tuberculosis infection. In regions where other MTBC subspecies are the primary pathogen among the cattle population, *M bovis* prevalence might substantially underestimate the burden of zoonotic tuberculosis. The operational definition of zoonotic tuberculosis provided by WHO, the World Organisation for Animal Health, and the Food and Agriculture Organization for the United Nations should be broadened to include other MTBC members capable of causing human disease such as *M orygis*. This research highlights the importance of a One Health approach to tuberculosis control in India, and elsewhere, in which medical surveys should be informed by corresponding veterinary data.

## Methods

### Study design and isolates

In this molecular epidemiological surveillance study, we used 940 positive mycobacteria growth indicator tube (MGIT) cultures previously collected at Christian Medical College in Vellore, India between Oct 1, 2018, and March 31, 2019. This study took place from Jan 5 to June 9, 2019. Institutional review board approval (approval number 11725, dated Dec 19, 2018) and ethical clearance from Christian Medical College were obtained for this study. All patient data were denominalised before inclusion of each isolate in our study. An isolate was defined as a pure pathogen collected from an individual patient. A sample was defined as the patient specimen that was previously cultured to obtain an isolate.

All PCR screening was done at Christian Medical College. WGS was done at Lala Lajpat Rai University of Veterinary and Animal Sciences in Hisar, India. WGS analysis and construction of phylogenetic trees was done at The Pennsylvania State University, State College, PA, USA, in collaboration with the United States Department of Agriculture in Ames, IA, USA. The study design and procedure are outlined in appendix 1 (p 1).

The MGIT cultures screened in this study were obtained from patients who visited the outpatient department at Christian Medical College with signs and symptoms of suspected tuberculosis (from Oct 1, 2018, to March 31, 2019). Pulmonary isolates were defined as those collected from the lung fluid or tissue; extrapulmonary isolates were defined as those collected from tissue other than the lungs. These definitions served to differentiate disseminated disease from disease confined to the lungs and their adjacent structures.

#### PCR assays

Cultures were screened with PCR assays previously established and validated at McGill University in Montreal, QC, Canada. DNA was prepared for screening by boiling.

We planned to screen the first 600 isolates by conventional PCR between Jan 5 and Jan 25, 2019, to assess the proportion of *M tuberculosis,* as opposed to *M bovis, M orygis*, or other MTBC subspecies. Two deletion-based conventional PCR assays were developed for this screening. A three-primer PCR was designed to detect the presence or absence of region of difference (RD) 9, which is present in *M tuberculosis* and absent from other MTBC subspecies (appendix 1 p 2).^11^ A six-primer PCR was developed to detect differences in the deletion size of RD12. The RD12 region is present in *M tuberculosis* and absent from *M bovis* and *M orygis*; however, the *M orygis* deletion is larger than in *M bovis* and the insertion sequence IS6110 is inserted (appendix 1 p 2).^11^ All primers were designed with Primer3 software (version 0.4.0; appendix 1 p 3).^15^ PCR reaction conditions are described in appendix 1 (p 4). PCR products were separated by gel electrophoresis on a 2% (w/v) agarose gel containing a 1:10 000 dilution of ethidium bromide.

A five-probe multiplex real-time (rt) PCR assay was developed to screen the remaining 340 isolates between May 11 and May 20, 2019. Forty isolates identified as *M tuberculosis* and all isolates identified as MTBC other than *M tuberculosis* by conventional PCR were also assessed by rtPCR to confirm consistent identity across assays. Four of the probes used have been described by Halse and colleagues,^16^ which detected the presence of RD1, RD9, RD12, and a conserved region external to RD9. The RD1 probe allows for the differentiation of wild-type *M bovis* from *M bovis* BCG.^11^ A fifth probe was designed to target the *M orygis*-specific single nucleotide polymorphism (SNP) in *Rv0444c* (G698C).^17^ These probes allowed for differentiation of several members of the MTBC (appendix 1 p 5). The *Rv0444c* probe and primers were designed with Primer Express (version 3.0). All primer and probe sequences, and conditions of the rtPCR reactions are given in appendix 1 (pp 3–4).

#### Sequencing assays

Isolates identified as MTBC other than *M tuberculosis* or as inconclusive by PCR were processed for WGS. Genomic DNA was extracted as previously described.^18^ Libraries were prepared with the Nextera DNA Flex Library Prep Kit (Illumina, San Diego, CA, USA). We assessed the quality of each library with a Fragment Analyzer Automated CE System (Agilent, Santa Clara, CA, USA) using a Next Generation Sequencing Fragment Kit (1–6000 bp; Agilent). Libraries were paired-end sequenced on an Illumina MiSeq System with a MiSeq Reagent Kit (version 3, 600-cycle). Sequences were deposited for download in the National Center for Biotechnology Information (NCBI) Sequence Read Archive (SRA) accession number SRP226525 (BioProject database number PRJNA575883).

For isolates not amplified by PCR, suggestive of a non-tuberculous mycobacteria, *hsp65* sequencing was done by the Sanger method, as previously described.^19^

### Bioinformatics

Genome sequences were assessed with the validate SNP (vSNP) tool of the US Department of Agriculture-Veterinary Services. The vSNP pipeline involved a two-step process. Step 1 determined SNP positions called against a best-matched reference (either *M tuberculosis* strain H37Rv or *M bovis* strain AF2122). Step 2 assessed SNPs called between related isolate groups, which shared a common SNP, to output SNP alignments, tables, and phylogenetic trees. For an SNP to be considered in a group there needed to be at least one locus with an allele count of 2, a quality score greater than 150, and map quality greater than 56, as per standard thresholds in vSNP. The output SNP alignment was used to assemble a maximum likelihood phylogenetic tree with RAxML software (version 8.2; GTRCATI model).^20^ Additional details of the pipeline are provided in appendix 1 (pp 6–7).

To situate sequences generated in this study within the context of global MTBC diversity, we used SPAdes (version 3.14.1) to assemble 373 genomes representing recognised lineages associated with humans and animals, derived from the NCBI SRA database, and constructed a phylogenetic tree with kSNP3.0 (appendix 2 p 1).^21^ We followed the kSNP3.0 manual instructions using the kmer value calculated with the kchooser tool.

To compare the newly sequenced genomes with sequences from south Asia, we searched the NCBI SRA using the search terms (“*Mycobacterium bovis*” OR “*Mycobacterium tuberculosis*” OR “*Mycobacterium africanum*” OR “*Mycobacterium orygis*” OR “*Mycobacterium canetti*” OR “*Mycobacterium caprae*” OR “*Mycobacterium bovis* BCG” NOT “H37Rv” NOT “H37Ra”) from (“India” OR “Bangladesh” OR “Nepal” OR “Sri Lanka” OR “Pakistan”). All sequences were downloaded from the SRA with the fasterq-dump tool from the SRA toolkit (version 2.9.6) and sequences were filtered for quality, according to the criteria in appendix 1 (p 8). Sequences of the required quality were run through vSNP (appendix 2 p 2). Phylogenetic trees were constructed with vSNP to compare the sequences from this study with the genomes from south Asia. SRA reference sequences of the MTBC lineages were also included for comparison (appendix 2 p 3). Phylogenetic trees were rooted to *M tuberculosis* strain H37Rv. As a post-hoc assessment, to determine whether human *M orygis* isolates were dispersed among cattle isolates, and whether cattle *M tuberculosis* isolates were dispersed among human isolates, sequences generated in this study were phylogenetically compared with *M orygis* sequences from south Asia and *M tuberculosis* sequences of cattle origin.

We visualised all phylogenetic trees using the interactive tree of life (version 4).^22^ Further details on tree assembly are provided in appendix 1 (pp 6–7).

### Statistical analysis

The minimum sample sizes were determined to be 300 pulmonary isolates and 300 extrapulmonary isolates to ensure that a zero numerator would indicate a 95% CI of 1·0% or less. Statistical analysis was done with GraphPad Prism (version 8.1.2). Prevalence ratios of extrapulmonary and pulmonary tuberculosis, as a function of age and mycobacterial species, were calculated in GraphPad and compared with Fisher's exact tests, with a p value of less than 0·05 considered to indicate statistical significance. Prevalence ratio 95% CIs were calculated with the Koopman asymptotic score in GraphPad.

### Role of the funding source

The funders of the study had no role in data collection, data analysis, or data interpretation. NJu is the Senior Program Officer at the Bill & Melinda Gates Foundation and was involved in study design and writing of the report. The corresponding authors had full access to all the data in the study and had final responsibility for the decision to submit for publication.

## Results

**Figure 1:**
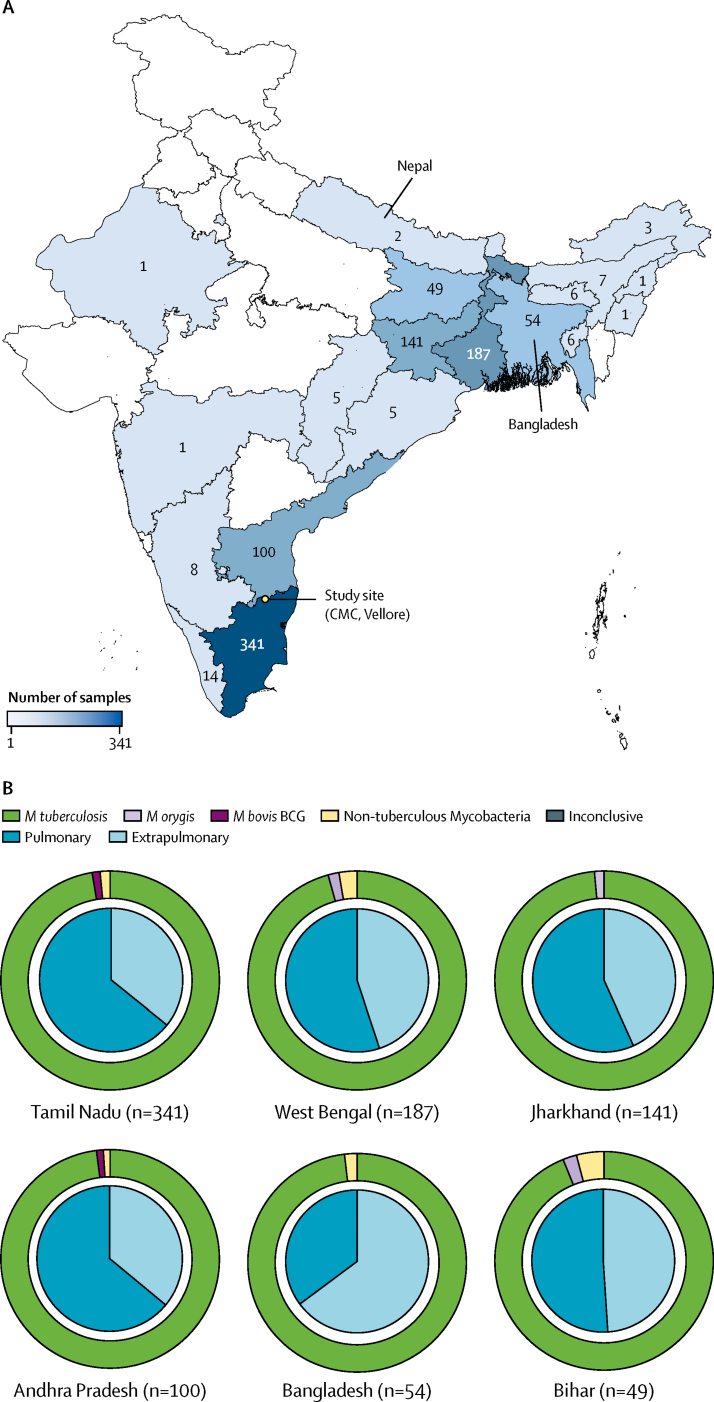
Distribution and sample types of patient isolates within India and surrounding countries (A) Geographical distribution of the collected isolates. Numbers indicate isolates collected per area. No isolates were screened from locations in white. (B) Sample types of isolates from locations where 20 or more samples were collected. Inner pie charts show the proportion of pulmonary and extrapulmonary isolates collected from each location. Outer doughnut charts indicate the proportion of mycobacterial subspecies collected from each location. Exact numbers are provided in appendix 3. CMC=Christian Medical College. *M tuberculosis*=*Mycobacterium tuberculosis. M orygis=Mycobacterium orygis. M bovis*=*Mycobacterium bovis*.

DNA was prepared from 940 positive MGIT cultures. In total, 548 pulmonary samples and 392 extrapulmonary samples were included in our study. Table 1 describes the characteristics of patients from whom isolates were obtained. Pulmonary isolates were from sputum (504 samples), pleural samples (30), lung biopsies (eight), endotracheal aspirate (three), bronchoalveolar lavage (two), and chest wall abscess (one). The extrapulmonary sample types are listed in appendix 1 (p 9). Extrapulmonary tuberculosis was most prevalent in patients of younger age. Extrapulmonary isolates were obtained from 19 (73·1%) of 26 patients younger than 10 years, and 373 (40·8%) of 914 patients aged 10 years and older (prevalence ratio 1·8 [95% CI 1·3–2·2]; p=0·0018). Meanwhile, pulmonary tuberculosis was most prevalent in patients of older age. Pulmonary isolates were obtained from 88 (80·0%) of 110 patients aged 60 years and older, and 460 (55·4%) of 830 patients younger than 60 years (prevalence ratio 1·4 [1·3–1·6]; p<0·0001). Most patients were from 20 states and territories in India (884 [94%] of 940); all other patients were from Bangladesh (54 [6%]) and Nepal (2 [<1%]; table 1, figure 1A). Within India, most isolates were collected from patients living in the states of Tamil Nadu (341 [39%] of 884 Indian isolates), West Bengal (187 [21%]), and Andhra Pradesh (100 [11%]). Pulmonary isolates comprised the majority of isolates from each location, except for Bangladesh where 35 (64·8%) of 54 isolates were extrapulmonary (figure 1B).

**Table 1:** Characteristics of patients from whom isolates were obtained and screened for zoonotic tuberculosis

		**Pulmonary sample (n=548 patients)**	**Extrapulmonary sample (n=392 patients)**	**Total (n=940 patients)**
**Age, years**				
0–9		7 (1%)	19 (5%)	26 (3%)
10–19		54 (10%)	42 (11%)	96 (10%)
20–29		112 (20%)	101 (26%)	213 (23%)
30–39		100 (18%)	83 (21%)	183 (19%)
40–49		99 (18%)	71 (18%)	170 (18%)
50–59		88 (16%)	54 (14%)	142 (15%)
60–69		64 (12%)	14 (4%)	78 (8%)
≥70		24 (4%)	8 (2%)	32 (3%)
**Sex**				
Female		185 (34%)	178 (45%)	363 (39%)
Male		363 (66%)	214 (55%)	577 (61%)
**Location**				
India		528 (96%)	356 (91%)	884 (94%)
	South	297 (56%)	168 (47%)	465 (53%)
	East	209 (40%)	173 (49%)	382 (43%)
	Northeast	15 (3%)	13 (4%)	28 (3%)
	Central	5 (1%)	0	5 (1%)
	North	2 (<1%)	1 (<1%)	3 (<1%)
	West	0	1 (<1%)	1 (<1%)
Bangladesh		19 (3%)	35 (9%)	54 (6%)
Nepal		1 (<1%)	1 (<1%)	2 (<1%)

Data are number of patients (%). Percentages do not always equal 100% due to rounding. For the purposes of the present study, regions of India are divided by location as follows: south India includes the Andaman and Nicobar Islands, Andhra Pradesh, Karnataka, Kerala, Lakshadweep, Puducherry, Tamil Nadu, and Telangana; east India includes West Bengal, Bihar, Jharkhand, and Odisha; northeast India includes Arunachal Pradesh, Assam, Manipur, Meghalaya, Mizoram, Nagaland, Sikkim, and Tripura; central India includes Chhattisgarh and Madhya Pradesh; north India includes Jammu and Kashmir, Himachal Pradesh, Punjab, Chandigarh, Uttarakhand, Haryana, National Capital Territory of Delhi, Rajasthan, and Uttar Pradesh; and west India includes Dadra and Nagar Haveli, Daman and Diu, Goa, Gujarat, and Maharashtra.

Isolates were assessed by conventional and rtPCR to evaluate the proportions of MTBC subspecies. Following PCR screening, 25 isolates were selected for WGS (appendix 1 p 10). Based on PCR, these isolates were provisionally identified as *M orygis* (seven)*, M bovis* BCG (six), inconclusive (eight), and *M tuberculosis* isolates negative for RD12 (two). The two remaining isolates, identified as *M tuberculosis*, were assigned for WGS in error. Of the eight inconclusive isolates, seven were categorised as such because of delayed amplification of the RD12 probe, and one was selected following poor amplification of the RD1 probe.

The proportions of mycobacterial species identified in our study after all genotyping are described in table 2 and appendix 3. No wild-type *M bovis* was identified. Seven (0·7%) of the 940 isolates were identified as *M orygis*, six of which were extrapulmonary. *M orygis* was enriched in extrapulmonary isolates compared with pulmonary isolates, with a prevalence ratio of 8·4 (95% CI 1·3–52·9; p=0·023). Six of the seven *M orygis* isolates were from patients from Bihar, Jharkhand, and West Bengal in northeast India (figure 1B, appendix 3). The remaining *M orygis* isolate was from a patient from Karnataka in south India. 913 isolates (97·1%) were identified as *M tuberculosis.* The two isolates that lacked RD12 on PCR were assigned as *M tuberculosis* and not *Mycobacterium canettii* by WGS. The seven isolates categorised as inconclusive because of delayed amplification of the RD12 probe contained an SNP in the RD12 reverse primer sequence, and were assigned as *M tuberculosis*. The other isolate categorised as inconclusive because of poor amplification of RD1 was found to have a 5962 bp deletion spanning Rv3871–Rv3877 including the site of the RD1 probe, and was assigned as *M tuberculosis*. Five isolates (0·5%) were confirmed as *M bovis* BCG by WGS. All of these isolates were identified in patients aged 3 years or younger, consistent with expectations for a population in which BCG vaccine is routinely administered. 15 isolates (1·6%) were identified as non-tuberculous mycobacteria. The most common non-tuberculous species were *Mycobacterium abscessus* and *Mycobacterium intracellulare* (three isolates each; appendix 1 p 11). The identification of one isolate was inconsistent across methods, suggestive of tube mislabelling: the isolate was identified as *M bovis* BCG by rtPCR and *M tuberculosis* by WGS, and was assigned as *M tuberculosis*.

**Table 2:** Isolate subspecies determined by PCR and WGS

	**Pulmonary isolates (n=548)**	**Extrapulmonary isolates (n=392)**	**Total isolates (n=940)**
*Mycobacterium tuberculosis*	535 (97·6%)	378 (96·4%)	913 (97·1%)*
*Mycobacterium orygis*	1 (0·2%)	6 (1·5%)	7 (0·7%)†
*Mycobacterium bovis*	0	0	0
*Mycobacterium bovis* BCG	1 (0·2%)	4 (1·0%)	5 (0·5%)†
Non-tuberculous mycobacteria	11 (2·0%)	4 (1·0%)	15 (1·6%)

WGS=whole-genome sequencing.

* Including two *M tuberculosis* isolates negative for RD12 on PCR but confirmed by WGS (both extrapulmonary); eight inconclusive isolates on PCR identified as *M tuberculosis* by WGS (six pulmonary, two extrapulmonary); and one pulmonary isolate identified as *M bovis* BCG by PCR and *M tuberculosis* by WGS.

† All detected by PCR and confirmed by WGS.

**Figure 2:**
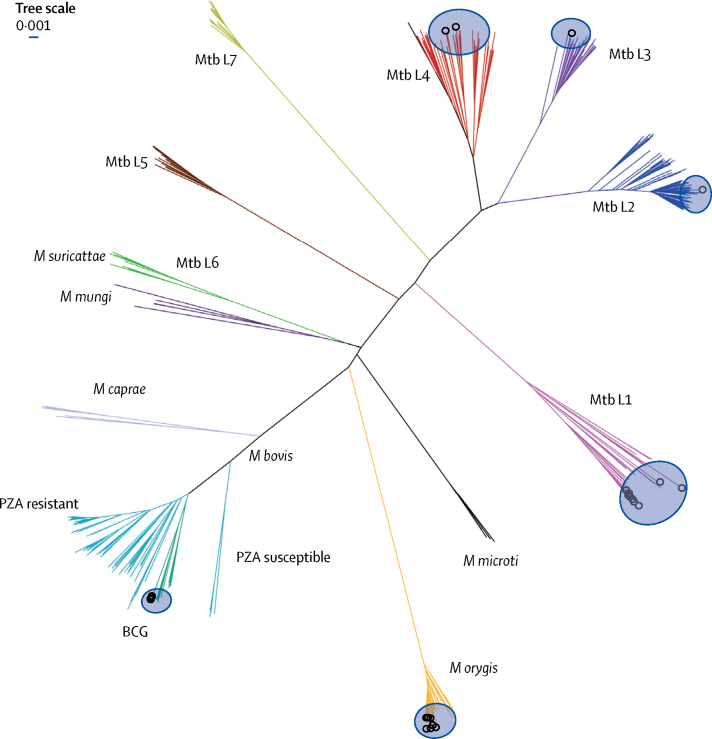
Phylogenies of newly sequenced isolates in the context of genetic diversity among global MTBC isolates The unrooted tree shows clustering of 25 isolates sequenced in this study (circles) in the context of representative samples (n=373) of MTBC lineages from around the world (appendix 2 p 1). MTBC=*Mycobacterium tuberculosis* complex. Mtb L1=*Mycobacterium tuberculosis* lineage 1. *M suricattae*=M*ycobacterium* suricattae. *M mungi*=M*ycobacterium mungi. M caprae*=M*ycobacterium caprae. M microti*=M*ycobacterium microti. M bovis*=M*ycobacterium bovis*.PZA=pyrazinamide. *M orygis*=M*ycobacterium orygis*.

A phylogenetic tree was constructed of the 25 isolates sequenced in this study in the context of the MTBC lineages circulating worldwide (figure 2). The 13 *M tuberculosis* isolates were dispersed across *M tuberculosis* lineage 1 (Mtb L1; nine isolates), Mtb L2 (one), Mtb L3 (one), and Mtb L4 (two). Of the nine Mtb L1 genomes collected in our study, seven were found to cluster together; these seven isolates shared an SNP in the RD12 reverse primer sequence. All five confirmed *M bovis* BCG isolates identified as the Russian strain. No isolates clustered with wild-type *M bovis* or *M caprae*. The seven isolates that clustered with previously sequenced *M orygis* isolates were separated from each other by 66–282 SNPs (appendix 1 p 12).

**Figure 3:**
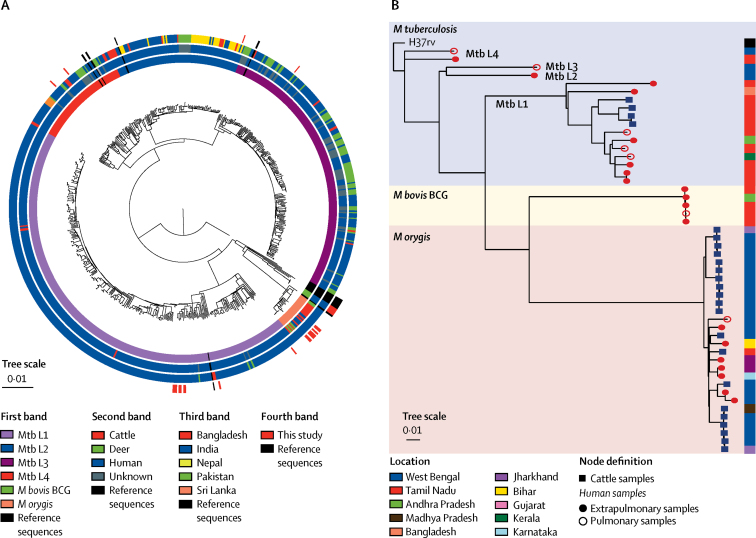
Phylogenies of newly sequenced isolates in the context of *Mycobacterium tuberculosis* complex isolates in south Asia (A) The outermost band shows our 25 sequenced isolates in the context of south Asian sequences. Other bands represent 715 MTBC sequences downloaded from the National Center for Biotechnology Information Sequence Read Archive. The sequences represent different lineages (first band from the middle), different host species (second band), and different countries (third band). Cattle isolates included one bison isolate as part of the Bovidae family. (B) Represented in the dendrogram are isolates from this study, all downloaded *M orygis* sequences (all of cattle origin; 20 sequences), and all downloaded Mtb L1 sequences of cattle origin (four). One sample was from Bangladesh and the rest were from Indian states. *M tuberculosis*=*Mycobacterium tuberculosis*. Mtb L1=*M tuberculosis* lineage 1. *M bovis*=*Mycobacterium bovis. M orygis*=*Mycobacterium orygis*.

A search of the NCBI SRA for MTBC isolates from south Asia generated 1640 genomes. These sequences were filtered before tree assembly, giving 715 high-quality sequences (appendix 2 p 2). The downloaded genomes were phylogenetically compared with our newly sequenced isolates and their respective metadata (figure 3A, appendix 2 p 4). No wild-type *M bovis* was identified in the 715 genomes. As expected, Mtb L1 and Mtb L3 were enriched. Almost all Mtb L1 genomes were from India (two sequences were from Bangladesh and three were from Pakistan); whereas, the Mtb L2, L3, and L4 sequences were from a variety of other countries in south Asia. Sequences of cattle origin from south Asia were represented in Mtb L1 (four isolates) and the *M orygis* lineage (20 isolates). On post-hoc assessment, the four isolates of cattle origin in Mtb L1 were dispersed among human sequences; similarly, the seven human *M orygis* isolates from this study were dispersed among sequences collected from cattle (figure 3B).

## Discussion

Zoonotic tuberculosis is increasingly recognised as a potential threat to the control of tuberculosis.^23^ *M bovis* was identified as a cause of bovine tuberculosis in 1898, nearly a century before descriptions of other distinct MTBC subspecies infecting cattle, at which point the scientific community had arrived at a general consensus that zoonotic risks associated with tuberculosis were caused by *M bovis.*^24^ *M bovis* as the causal agent continues to be defined as zoonotic tuberculosis by WHO, the World Organisation for Animal Health, and the Food and Agriculture Organization of the United Nations,^1^, ^3^, ^4^ despite mounting evidence that human infections can also be caused by other members of the MTBC, such as *M orygis*.^11^, ^12^, ^13^, ^14^, ^25^ To evaluate *M bovis* as a proxy for zoonotic tuberculosis and investigate the potential role of other MTBC subspecies, we did a molecular epidemiological screen of 940 clinical tuberculosis isolates from a large referral hospital in southern India. Our search was then broadened to include 715 MTBC sequences from south Asia deposited in the SRA database. Notably, no wild-type *M bovis* was identified in our study. Seven cases of tuberculosis were found to be caused by *M orygis,* and these were more likely to present as extrapulmonary tuberculosis (table 2). These findings suggest that *M bovis* might be an inadequate proxy for zoonotic tuberculosis infection in regions where *M bovis* is not the predominant MTBC member in livestock.

Detection of *M orygis* in this study is concordant with previous reports linking *M orygis* to patients with tuberculosis from south Asia.^11^, ^12^, ^13^, ^14^ Marcos and colleagues^12^ identified eight *M orygis* isolates in individuals originating from India, Pakistan, or Nepal. All seven *M orygis* isolates collected by Lavender and colleagues^13^ were from patients from India. Additionally, *M orygis* has been detected in cattle and rhesus monkeys in Bangladesh.^11^, ^14^ Collectively these data indicate that members of the MTBC complex other than *M bovis* might be relatively more prevalent in livestock in these countries. In 2018, Brites and colleagues^26^ proposed that the distribution of *M orygis* and *M bovis* could be traced back to two independent cattle domestication events, wherein M orygis became a pathogen of cattle in south Asia, primarily Bos indicus, while M bovis became a pathogen of Bos taurus. Virulence studies comparing pathogenicity between these two subspecies in infected cattle breeds are yet to be done. Conversely, Rahim and colleagues^14^ suggested that the global distribution of these subspecies indicates the emergence of *M orygis* before *M bovis* from a common MTBC ancestor, wherein *M orygis* might have been dispersed to south Asia with the migration of humans and become established before the arrival of *M bovis.* However, previous establishment of an MTBC lineage does not negate the potential for introduction of another lineage at a later point in time, as shown by the widespread dispersal of Mtb L2 and L4.^27^

We observed *M tuberculosis* isolates from south Asia to be dispersed across lineages 1–4 with an enrichment of Mtb L1 and L3, concordant with previous reports.^27^ MTBC sequences of cattle origin from south Asia were distributed among lineages Mtb L1 and *M orygis*, highlighting the need to understand pathogenicity and transmission dynamics of these pathogens in cattle. *M tuberculosis* transmission from humans to cattle has been reported, which might have important implications for transmission control in countries with a high burden of endemic tuberculosis.^28^, ^29^ Collectively, our data support the occurrence of zoonotic tuberculosis in India but suggest an association with *M orygis*, and possibly *M tuberculosis*, rather than *M bovis.*

In this molecular epidemiological study we screened almost 1000 clinical tuberculosis isolates from positive MGIT cultures. Our approach has several advantages compared with previous studies.^8^, ^9^, ^10^ The use of positive broth cultures ensured that active tuberculosis cases were microbiologically confirmed, unlike PCR-based investigations done on direct clinical samples without culture corroboration. The two-step protocol enabled provisional identification by PCR and assessment of inconclusive results by WGS. In addition, compared with conventional PCR, our five-probe multiplex rtPCR allowed for streamlined differentiation of many MTBC subspecies in a single reaction (appendix 1 p 5), showing promise for easy adoption in routine screening for zoonotic tuberculosis, depending on the needs and resources of the location. Furthermore, the detection of *M bovis* BCG served as a positive control for the PCR assays to identify *M bovis*, were it present. However, deletion of RD1, along with WGS analysis, confirmed that these isolates were the Russian BCG strain, consistent with the vaccine currently used in India. An important limitation is our single-centre surveillance approach, meaning the isolates collected might be biased to locations within or nearby Tamil Nadu or to the patients who travelled to the study site. Future studies need to be done in other areas of India and other countries in south Asia to obtain a representative dataset. Furthermore, WGS analysis indicated that RD12 might be an inadequate marker for detection of *M tuberculosis*. This region was deleted in two *M tuberculosis* isolates, and seven *M tuberculosis* isolates were first categorised as inconclusive due to an SNP in the RD12 reverse primer sequence.

In conclusion, this study indicates that *M bovis* might be uncommon in India, and thus its detection is potentially an ineffective proxy for the prevalence of zoonotic tuberculosis. The operational definition of zoonotic tuberculosis should be broadened to include other MTBC subspecies capable of causing human disease. The increasing evidence supporting *M orygis* endemicity in south Asia and the identification of *M tuberculosis* in cattle highlight the importance of a One Health approach, involving multisectoral collaboration across the veterinary and clinical sectors, to the control of tuberculosis in India.
