# Supplementary material for: Reconsidering Mycobacterium bovis as a proxy for zoonotic tuberculosis: a molecular epidemiological surveillance study
Source: Lancet Microbe. 2020 Jun;1(2):e66–73. doi: 10.1016/S2666-5247(20)30038-0 (PMC7325494; doi:10.1016/S2666-5247(20)30038-0)
Supplement: Supplementary appendix 1 [file mmc1.pdf]

THE LANCET
Microbe

Supplementary appendix

This appendix formed part of the original submission and has been peer reviewed. We post it as supplied by the authors.

Supplement to: Duffy S C, Srinivasan S, Schilling M A, et al. Reconsidering *Mycobacterium bovis* as a proxy for zoonotic tuberculosis: a molecular epidemiological surveillance study. *Lancet Microbe* 2020; **1:** e66–73.

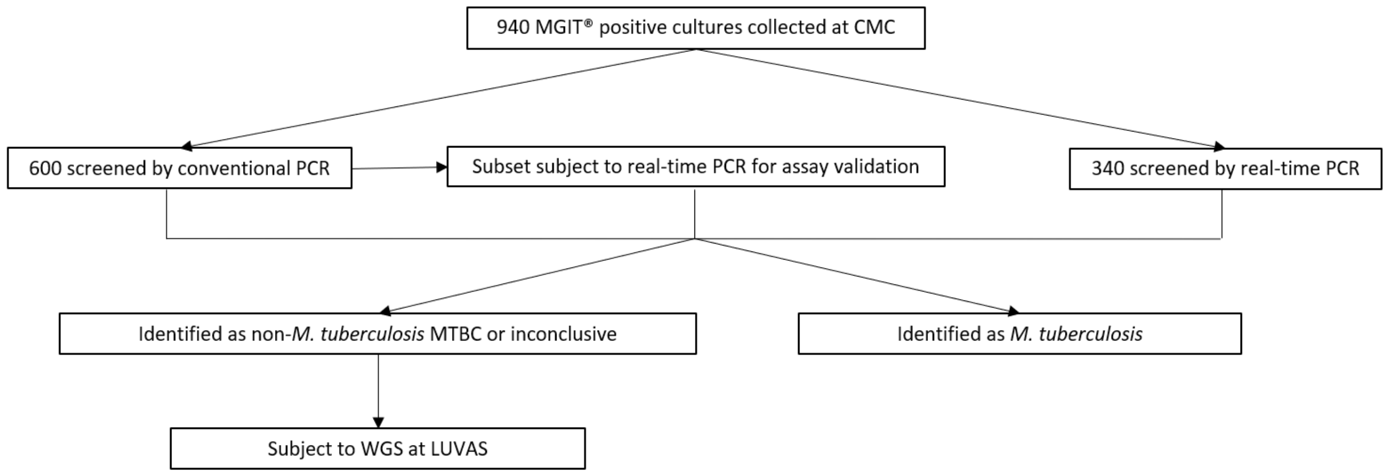

***Supplementary Figure 1:* Study design**

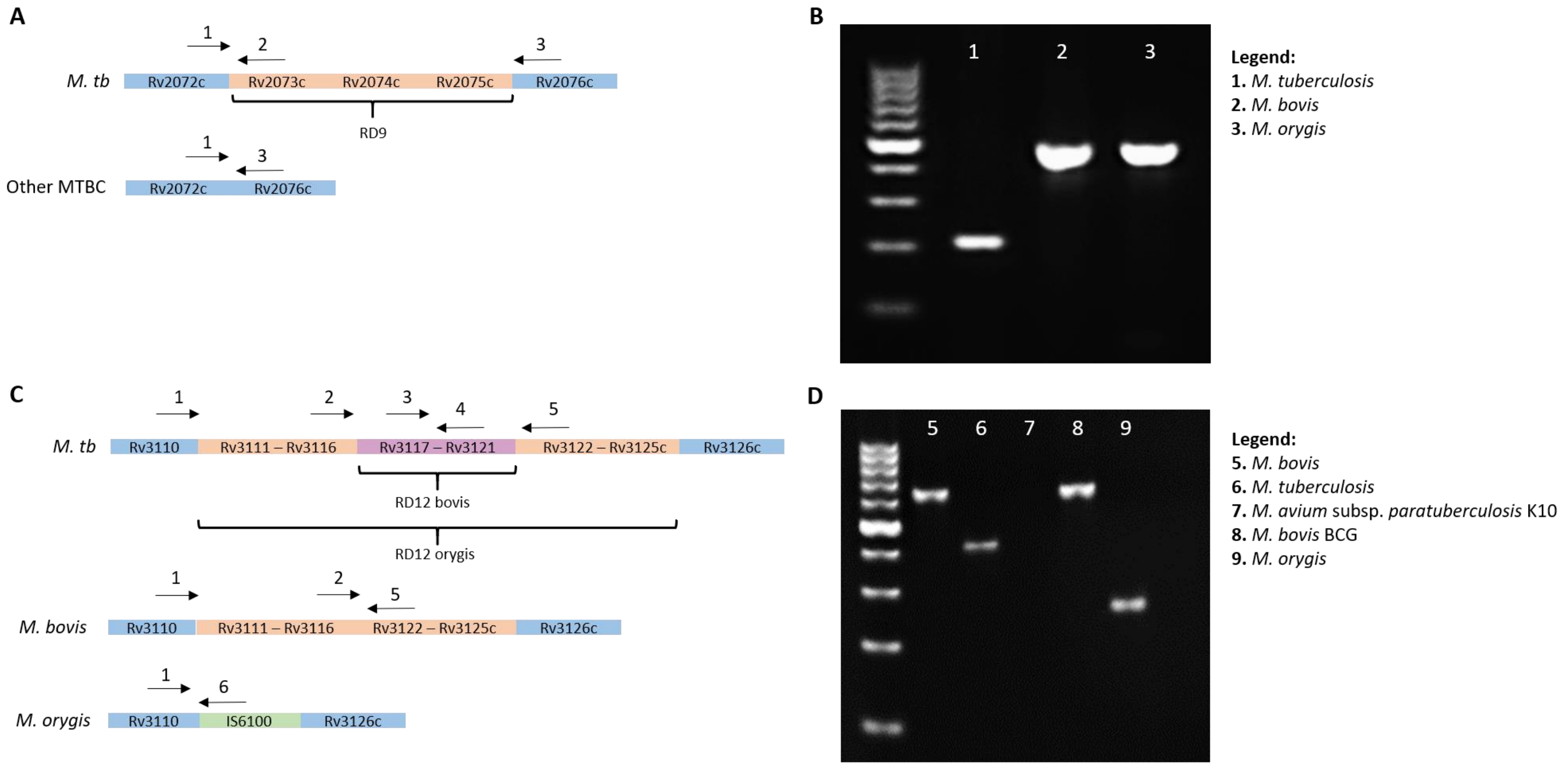

Supplementary Figure 2: **Conventional PCR to detect differences in deletions of RD9 and RD12**
SFig 2A: A three-primer PCR reaction was developed to detect the presence of absence of RD9, which is found in *M. tb* but is absent in all other MTBC members (van Ingen *et al*. 2012). **SFig 2B:** A 209bp band is amplified when *M. tb* is present and a 410bp band is amplified when other MTBC members are present. **SFig 2C:** A six-primer PCR was developed to detect differences in the deletion size of RD12. RD12 is present in *M. tb* and absent in *M. bovis* and *M. orygis.* In *M. orygis,* the RD12 deletion is larger and is replaced with IS6100 (van Ingen *et al*. 2012). **SFig 2D:** A 409bp band is amplified when *M. tb* is present, a 615bp band is amplified when *M. bovis* or BCG is present, and a 264bp band is amplified when *M. orygis* is present.

Assay	Name	Sequence (5' → 3')	Dye	Quencher
Conventional PCR	RD9_Forward	CCGATACCATGCAACAACGG		
	RD9_Reverse1	CGGTCTCTCCGAGCATTC		
	RD9_Reverse2	GCTCGAGCTAGACCTGCAC		
	RD12Mtb_Forward	GTATTTGCGCCCATATCCTGG		
	RD12Mtb_Reverse	CCTGGCTTCAAGCACCATTC		
	RD12Mbovis_Forward	GGCCATCAACGTCAAGAACCTC		
	RD12Mbovis_Reverse	CGAACTCGTATTTTGTGGCCAC		
	RD12Morygis_Forward	GTGGAAATGGAAGCGTTGACC		
	RD12Morygis_Reverse	GGTACCTCCTCGATGAACCAC		
Real-time PCR	Rv0444c_Probe	CTCGGCTGACCCGA	FAM	MGBNFQ
	Rv0444c_Forward	GATGCTGGGCACCATTGTC		
	Rv0444c_Reverse	GCCCACCGGTACCATCTTG		
	RD1 Probe	CACTCTGAGAGGTTGTCA	VIC	MGBNFQ
	RD1_Forward	CCCTTTCTCGTGTTTATACGTTTGA		
	RD1_Reverse	GCCATATCGTCCGGAGCTT		
	RD9 Probe	AGGTTTCA+CCTTCGAC+CC	TEX615	BHQ
	RD9_Forward	TGCGGGCGGACAACTC		
	RD9_Reverse	CACTGCGGTCGGCATTG		
	RD12 Probe	TGCGCTGACCCCAC	NED	MGBNFQ
	RD12_Forward	CGTTGGAACGCGAAATACG		
	RD12_Reverse	CCAGGATATGGGCGCAAAT		
	extRD9_Probe	G+TT+CTTCAG+CTGGT+CC	CY5	BHQ
	extRD9_Forward	GCCACCACCGACTCATAC		
	extRD9_Reverse	CGAGGAGGTCATCCTGCTCTA		

Supplementary Table 1: **Primer and probe sequences for conventional and real-time PCR assays**
The real-time PCR RD1, RD9, RD12, and ext-RD9 probes and primers are as described in Halse *et al*. The Rv0444c, RD1, and RD12 probes are Taqman MGB probes. The RD9 and ext-RD9 probes are locked nucleic acid probes. A '+' indicates insertion of a locked nucleic acid base.

Assay	Master mix	Thermocycling conditions
Conventional PCR	6.25 µl of 10X *Taq* buffer (Thermo Scientific)<br>6.25 µl acetamide 50% (wt/vol)<br>1.6 mM $MgCl_2$<br>0.2 mM deoxynucleoside triphosphates (dNTPs)<br>2.5 U per reaction *Taq* polymerase (Thermo Scientific)<br>500 nM of each primer<br>5µl of template DNA<br>Sterile water<br>50µl total volume	Initial denaturation 94°C for 3 minutes<br>35 cycles of:<br>- Denaturation at 94°C for 30 seconds<br>- Annealing at 55°C for 1 minute<br>- Elongation at 72°C for 1 minute,<br>Final elongation step at 72°C for 10 minutes
Real-time PCR	10 µl TaqMan multiplex master mix (Applied Biosystems)<br>450 nM of each primer<br>125 nM of each probe<br>1 µl of template DNA<br>Sterile water<br>20µl total volume	95°C for 10 minutes<br>40 cycles of:<br>- 95°C for 15s<br>- 60°C for 1 minute

Supplementary Table 2: **Master mix preparation and thermocycling conditions for conventional and real-time PCR assays**

	RD1	RD9	RD12	Rv0444c	Ext-RD9
M. tuberculosis	+	+	+	-	+
M. orygis	+	-	-	+	+
M. bovis	+	-	-	-	+
***M. bovis* BCG**	-	-	-	-	+
M. africanum	+	-	+	-	+
M. microti	-	-	+	-	+
**NTMs**	-	-	-	-	-

Supplementary Table 3: **Interpretation of RT-PCR results to determine MTBC sample identity**

Supplementary Methods

Bioinformatics

Sequences were assessed using the United States Department of Agriculture Animal and Plant Health Inspection Service Veterinary Services pipeline vSNP (https://github.com/USDA-VS/vSNP). The vSNP pipeline involved a two-step process. Step 1 determined SNP positions called within the sequence. Paired FASTQ files were processed using BWA-MEM to align reads to a reference genome *M. tuberculosis* H37Rv (NC_000962.3) for sequences included in this study ([1]). Duplicate reads were tagged and removed using the Mark Duplicates tool from Picard v 2.20.2 (http://broadinstitute.github.io/picard). SNPs were called using FreeBayes v. 1.3.1 ([2]). Unmapped reads shorter than 64 base pairs were removed and low-coverage contigs with an average k-mer coverage of less than 5 were removed. Depth of coverage was calculated using Pysam (https://github.com/pysam-developers/pysam) and positions with zero coverage were added to the VCF file. Step 2 assessed SNPs called between closely related isolate groups to output SNP alignments, tables and phylogenetic trees. For a SNP to be considered in a group there must have been at least one position with an allele count (AC) =2, quality score >150 and map quality > 56. Once determined, SNPs were aligned using the following workflow. If the quality score for a SNP position was greater than 150, the alternate allele was called if AC=2. However, if AC=1, the position was called ambiguous. Deletions were called when the alternate allele was a gap. If the quality score was between 50 and 150, the allele was marked N. If the quality score was less than 50, then the reference allele was called. Uninformative SNPs were not included. BAM files were used to visualize SNP calls. Unreliable positions due to read alignment error may have been removed from the analysis. The output SNP alignment was used to assemble a maximum likelihood phylogenetic tree using RAxML GTRCATI model ([3]).

Phylogenetic tree assembly

To compare the 25 newly sequenced genomes in the context of sequences from South Asia, a NCBI Sequence Read Archive (SRA) (https://www.ncbi.nlm.nih.gov/sra) search was performed using the search terms ("*Mycobacterium bovis*" OR "*Mycobacterium tuberculosis*" OR "*Mycobacterium africanum*" OR "*Mycobacterium orygis*" OR "*Mycobacterium canetti*" OR "*Mycobacterium caprae*" OR "*Mycobacterium bovis* BCG" NOT "H37Rv" NOT "H37Ra") from ("India" OR "Bangladesh" OR "Nepal" OR "Sri Lanka" OR "Pakistan"). This search yielded 1640 genomes. These sequences were then filtered prior to tree assembly (Supplementary Figure 3). A total of 215 were excluded because they were not from studies that performed with paired end sequencing. A further 103 were excluded as they did not contain forward and reverse read files. Another 589

sequences whose average depth of coverage was not between 60-400 were excluded. Finally, 18 were excluded after running vSNP step 2 due to the samples being mixed and generating multiple SNPs at all locations in the genome. In total, 715 sequences remained (Supplementary Table 5). All sequences were download from the SRA using the fasterq-dump tool from the sra toolkit v. 2.9.6 (https://ncbi.github.io/sra-tools/) and sequences were run through steps 1 and 2 of vSNP. Phylogenetic trees were constructed using vSNP to compare the 25 sequences from this study with the total 715 available genomes from South Asia and a subset. Reference sequences were also included for comparison (Supplementary Table 6). Phylogenetic trees were rooted to *M. tuberculosis* H37Rv. To compare the sequences collected in this study in the context of the global MTC, treeSPAdes (http://cab.spbu.ru/software/spades/) was used to assemble reads for kSNP3 ([4]). Genomes assemblies had expected complete genome sizes. The kSNP3 manual instructions were followed using kchooser calculated kmer value. All phylogenetic trees were visualized using the Interactive Tree of Life (iTOL) with their respective metadata (Supplementary Table 7) ([5]).

References:

1 Li H. Aligning sequence reads, clone sequences and assembly contigs with BWA-MEM. *arXiv e-prints* 2013; arXiv:1303.3997

2 Garrison E, Marth G. Haplotype-based variant detection from short-read sequencing. *arXiv e-prints* 2012; arXiv:1207.3907

3 Stamatakis A. RAxML version 8: A tool for phylogenetic analysis and post-analysis of large phylogenies. *Bioinformatics* 2014; **30**: 1312–3.

4 Gardner SN, Slezak T, Hall, BG. kSNP3.0: SNP detection and phylogenetic analysis of genomes without genome alignment or reference genomes. *Bioinformatics* 2015; **31**: 2877-8.

5 Letunic I, Bork P. Interactive Tree Of Life (iTOL) v4: recent updates and new developments. *Nucleic Acid Res* 2019; **47:** W256-9.

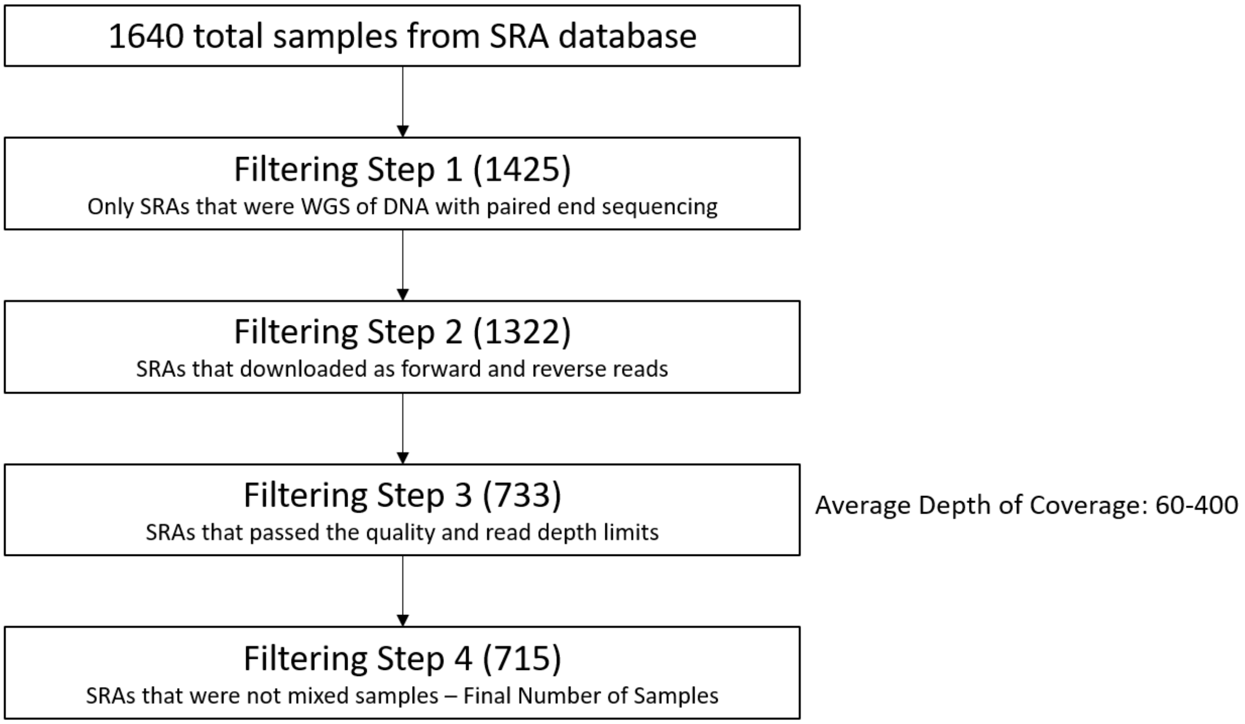

Supplementary Figure 3: **Selection and filtering pipeline of downloaded SRA MTBC genomes from South Asia**
A total of 1640 SRAs were downloaded from NCBI with the search terms ("*Mycobacterium bovis*" OR "*Mycobacterium tuberculosis*" OR "*Mycobacterium africanum*" OR "*Mycobacterium orygis*" OR "*Mycobacterium canetti*" OR "*Mycobacterium caprae*" OR "*Mycobacterium bovis* BCG" NOT "H37Rv" NOT "H37Ra") from ("India" OR "Bangladesh" OR "Nepal" OR "Sri Lanka" OR "Pakistan"). Through multiple filtering steps, the total number of sequences analyzed was 715.

Sample type	Number
Abdomen/Peritoneal fluid	15
Anal fistula	2
Biopsy	5
Bone	43
Bone marrow	2
Brain abscess	1
Colon	5
Cerebrospinal fluid	27
Fluid	5
Lymph node	162
Muscle abscess	5
Pus	56
Pericardium	1
Skin	1
Synovium	8
Tissue	40
Urine	13
Unspecified	1
Total	392

Supplementary Table 8: **Number and tissue types of extrapulmonary samples**
Extrapulmonary samples were defined as cultures from tissues other than the lungs or lung fluid.

Isolate number	Identification by real-time PCR	Average coverage	Genome coverage (%)	Phred quality score R1	Phred quality score R2	SNP count	Identification by WGS
P70	*M. bovis* BCG	103.2	99.35	34.6	31.1	803	*M. bovis* BCG Russia
P280	*M. bovis* BCG	80.4	99.15	34.5	31.1	1834	*M. tuberculosis* lineage 2
E50	*M. bovis* BCG	54.4	99.49	33.7	30.3	784	*M. bovis* BCG Russia
E110	*M. bovis* BCG	85.0	99.68	33.8	29.6	797	*M. bovis* BCG Russia
E280	*M. bovis* BCG	77.2	99.33	34.1	28.0	804	*M. bovis* BCG Russia
E396	*M. bovis* BCG	98.7	99.38	33.9	28.8	817	*M. bovis* BCG Russia
P326	Inconclusive	53.5	99.27	33.9	29.2	2322	*M. tuberculosis* lineage 1
P414	Inconclusive	82.2	99.42	33.6	27.8	2412	*M. tuberculosis* lineage 1
P448	Inconclusive	78.7	99.36	34.8	30.3	987	*M. tuberculosis* lineage 4
P465	Inconclusive	121.2	99.46	34.1	28.7	2476	*M. tuberculosis* lineage 1
E343	Inconclusive	277.6	99.53	35.1	32.5	2607	*M. tuberculosis* lineage 1
E369	Inconclusive	75.1	99.33	33.8	29.5	2429	*M. tuberculosis* lineage 1
E379	Inconclusive	115.2	99.51	34.2	29.3	2499	*M. tuberculosis* lineage 1
E415	Inconclusive	153.8	99.53	33.9	28.9	2510	*M. tuberculosis* lineage 1
E277	*M. tuberculosis*	138.6	99.39	34.8	30.4	1691	*M. tuberculosis* lineage 3
E428	*M. tuberculosis*	105.6	99.66	34.0	29.0	2594	*M. tuberculosis* lineage 1
P429	*M. orygis*	12.2	97.49	34.3	30.7	2299	*M. orygis*
E36	*M. orygis*	72.9	98.26	33.5	28.2	2547	*M. orygis*
E65	*M. orygis*	34.6	97.75	33.4	29.2	2408	*M. orygis*
E120	*M. orygis*	11.1	97.15	33.9	31.1	2330	*M. orygis*
E157	*M. orygis*	64.7	98.26	34.1	28.9	2478	*M. orygis*
E313	*M. orygis*	58.8	98.28	34.1	31.6	2483	*M. orygis*
E374	*M. orygis*	139.1	98.45	34.6	31.1	2566	*M. orygis*
E186	*M. tuberculosis* RD12 absent	90.3	98.74	34.1	29.1	968	*M. tuberculosis* lineage 4
E363	*M. tuberculosis* RD12 absent	107.4	99.00	34.5	30.2	2474	*M. tuberculosis* lineage 1

Supplementary Table 9: **Selection, library preparation and whole genome sequencing data of 25 selected isolates**

Sample name	PCR ID	Top BLAST match	Seq length	Query coverage	% Identity
E133	NTM	*Mycobacterium phocaicum*	430	100.00%	99.77%
E153	NTM	*Mycobacterium engbaekii*	440	99.00%	99.32%
E193	NTM	*Mycobacterium abscessus*	440	100.00%	100.00%
P24	NTM	*Mycobacterium sp. K328YA*	438	94.00%	100.00%
P30	NTM	*Mycobacterium alvei*	437	100.00%	98.40%
P390	NTM	*Mycobacterium abscessus*	432	100.00%	100.00%
P427	NTM	*Mycobacterium intracellulare*	419	100.00%	100.00%
P146	NTM	*Mycobacterium simiae*	443	99.00%	99.32%
P149	NTM	*Mycobacterium intracellulare*	424	100.00%	99.76%
E22	NTM	*Mycobacterium abscessus*	357	100.00%	99.72%
P219	NTM	*Mycobacterium yongonense*	359	100.00%	99.44%
P281	NTM	*Mycobacterium fortuitum*	362	100.00%	100.00%
P426	NTM	*Mycobacterium intracellulare*	333	100.00%	100.00%
P81	NTM	*Mycobacterium parascrofulaceum*	381	100.00%	99.48%

Supplementary Table 10: **Hsp65 sanger sequencing results of non-tuberculous mycobacteria (NTM) isolates**

	P429	E120	E36	E374	E313	E65	E157
P429	0	85.47	88.27	89.10	89.66	88.84	89.52
E120	282	0	86.30	87.55	88.13	87.27	87.84
E36	271	285	0	91.53	92.02	91.19	91.77
E374	252	259	214	0	93.29	92.38	93.07
E313	239	247	202	170	0	92.93	93.64
E65	258	265	223	193	177	0	97.39
E157	242	253	208	175	161	66	0

***Supplementary Figure 4*: SNP distances between 7 *M. orygis* isolates from this study**
The intensity of the color corresponds to the distance between isolates. The bottom portion of the matrix indicates the number of SNPs between the two isolates. The top portion indicates the percent of total SNPs shared between them

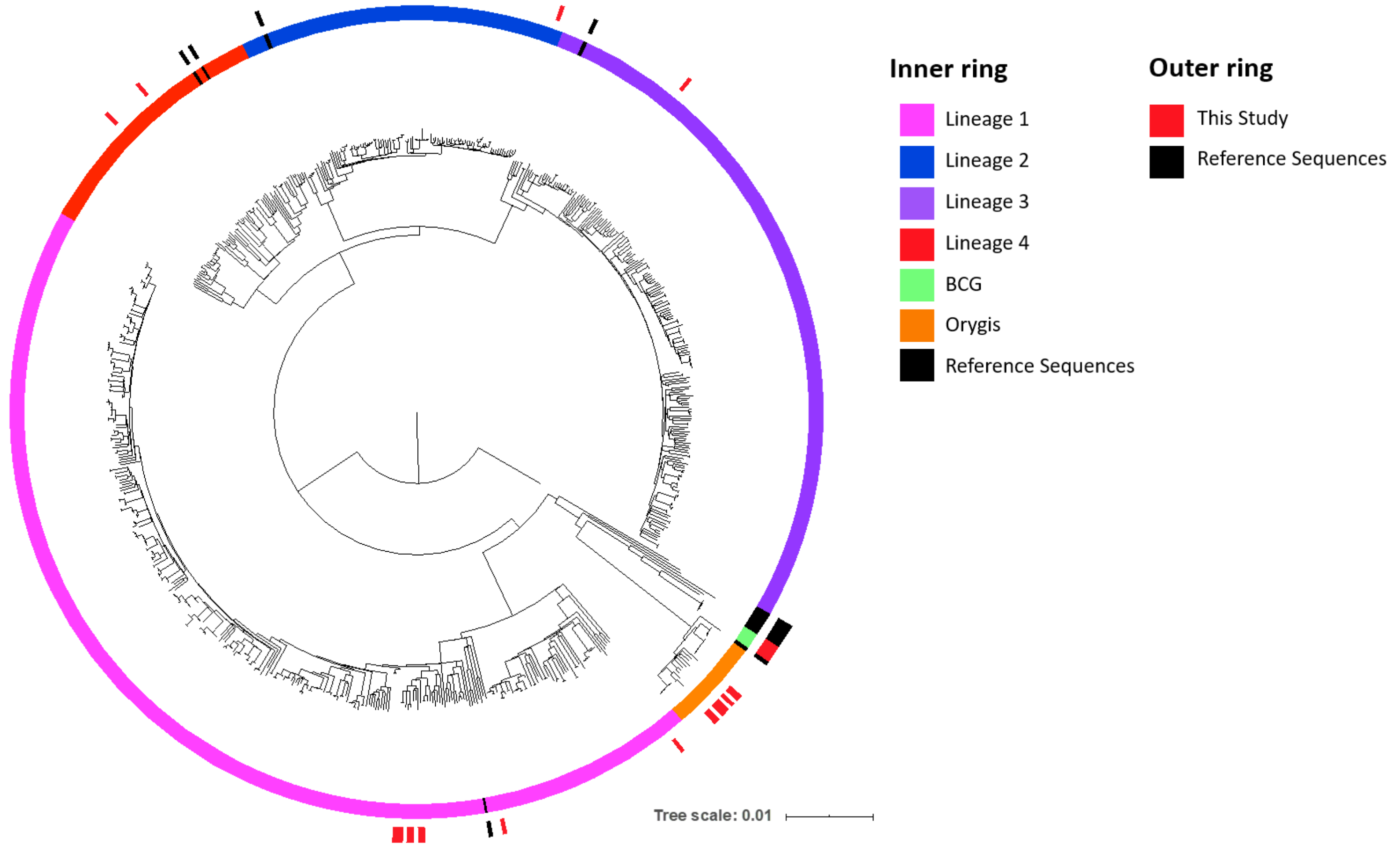

Supplementary Figure 5: **Maximum likelihood phylogenetic tree of newly sequenced isolates and 715 MTBC genomes collected from South Asia with lineage metadata**

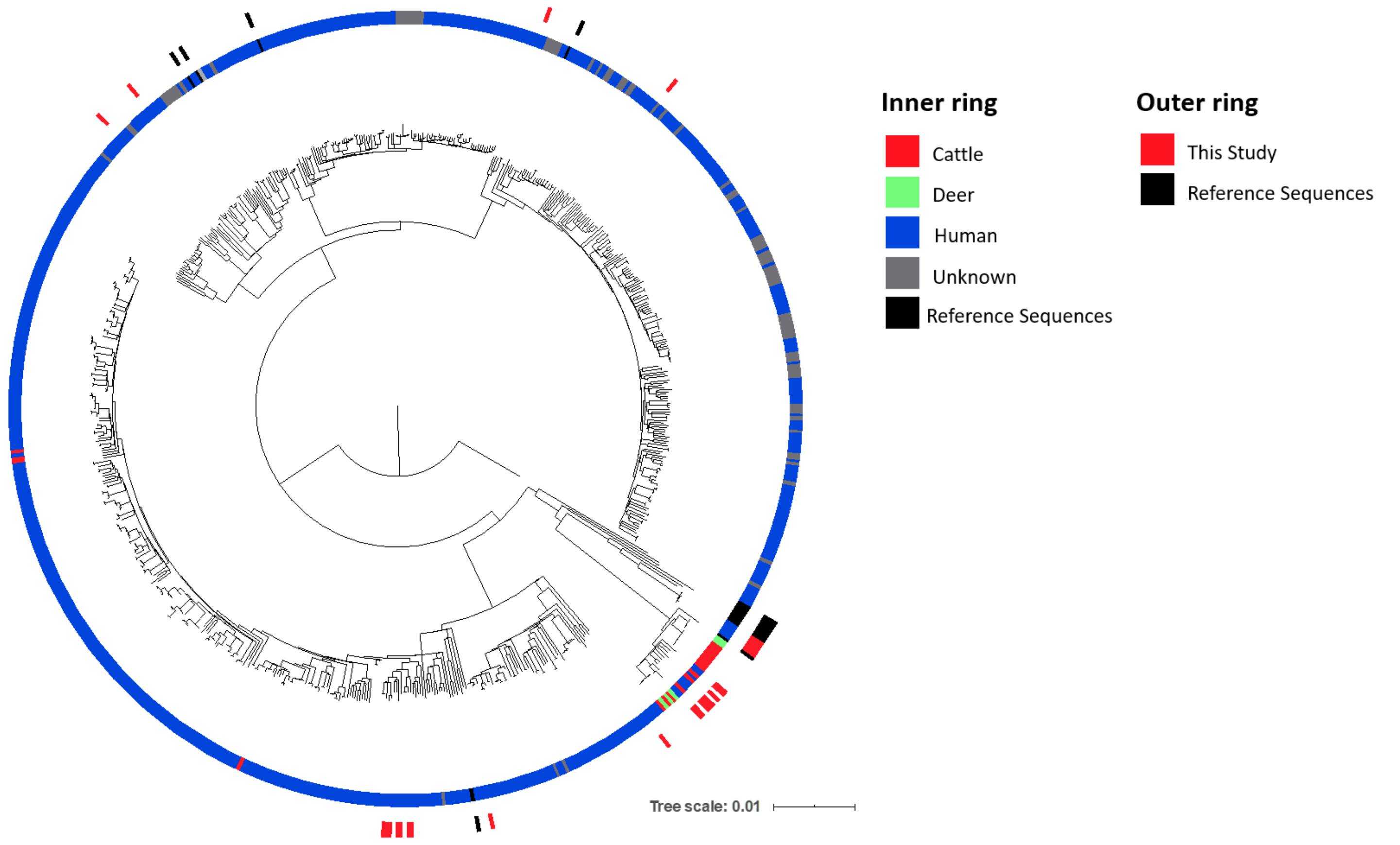

Supplementary Figure 6: **Maximum likelihood phylogenetic tree of newly sequenced isolates and 715 MTBC genomes collected from South Asia with host metadata**

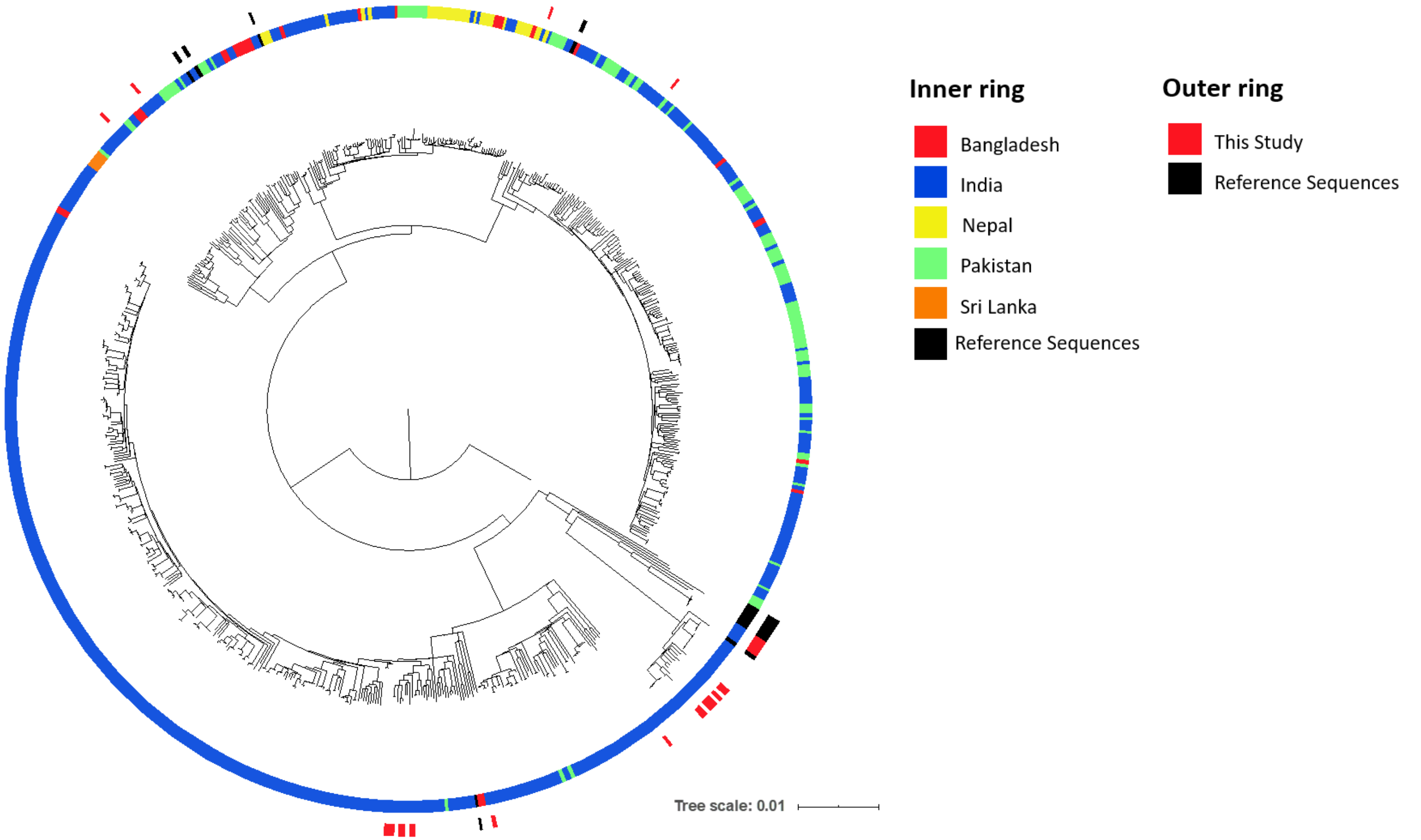

Supplementary Figure 7: **Maximum likelihood phylogenetic tree of newly sequenced isolates and 715 MTBC genomes collected from South Asia with country metadata**
